# ‘Neanderthal bone flutes’: simply products of Ice Age spotted hyena scavenging activities on cave bear cubs in European cave bear dens

**DOI:** 10.1098/rsos.140022

**Published:** 2015-04-01

**Authors:** Cajus G. Diedrich

**Affiliations:** PaleoLogic, Petra Bezruce 96, 26751 Zdice, Czech Republic

**Keywords:** Neanderthals, pseudo-bone flutes, Late Pleistocene cave bear dens, hyena scavenging, tooth marks, femur destruction stages

## Abstract

Punctured extinct cave bear femora were misidentified in southeastern Europe (Hungary/Slovenia) as ‘Palaeolithic bone flutes’ and the ‘oldest Neanderthal instruments’. These are not instruments, nor human made, but products of the most important cave bear scavengers of Europe, hyenas. Late Middle to Late Pleistocene (Mousterian to Gravettian) Ice Age spotted hyenas of Europe occupied mainly cave entrances as dens (communal/cub raising den types), but went deeper for scavenging into cave bear dens, or used in a few cases branches/diagonal shafts (i.e. prey storage den type). In most of those dens, about 20% of adult to 80% of bear cub remains have large carnivore damage. Hyenas left bones in repeating similar tooth mark and crush damage stages, demonstrating a butchering/bone cracking strategy. The femora of subadult cave bears are intermediate in damage patterns, compared to the adult ones, which were fully crushed to pieces. Hyenas produced round–oval puncture marks in cub femora only by the bone-crushing premolar teeth of both upper and lower jaw. The punctures/tooth impact marks are often present on both sides of the shaft of cave bear cub femora and are simply a result of non-breakage of the slightly calcified shaft compacta. All stages of femur puncturing to crushing are demonstrated herein, especially on a large cave bear population from a German cave bear den.

## 2. Introduction

### 2.1 First ‘bone flute descriptions’

**Figure 1.**
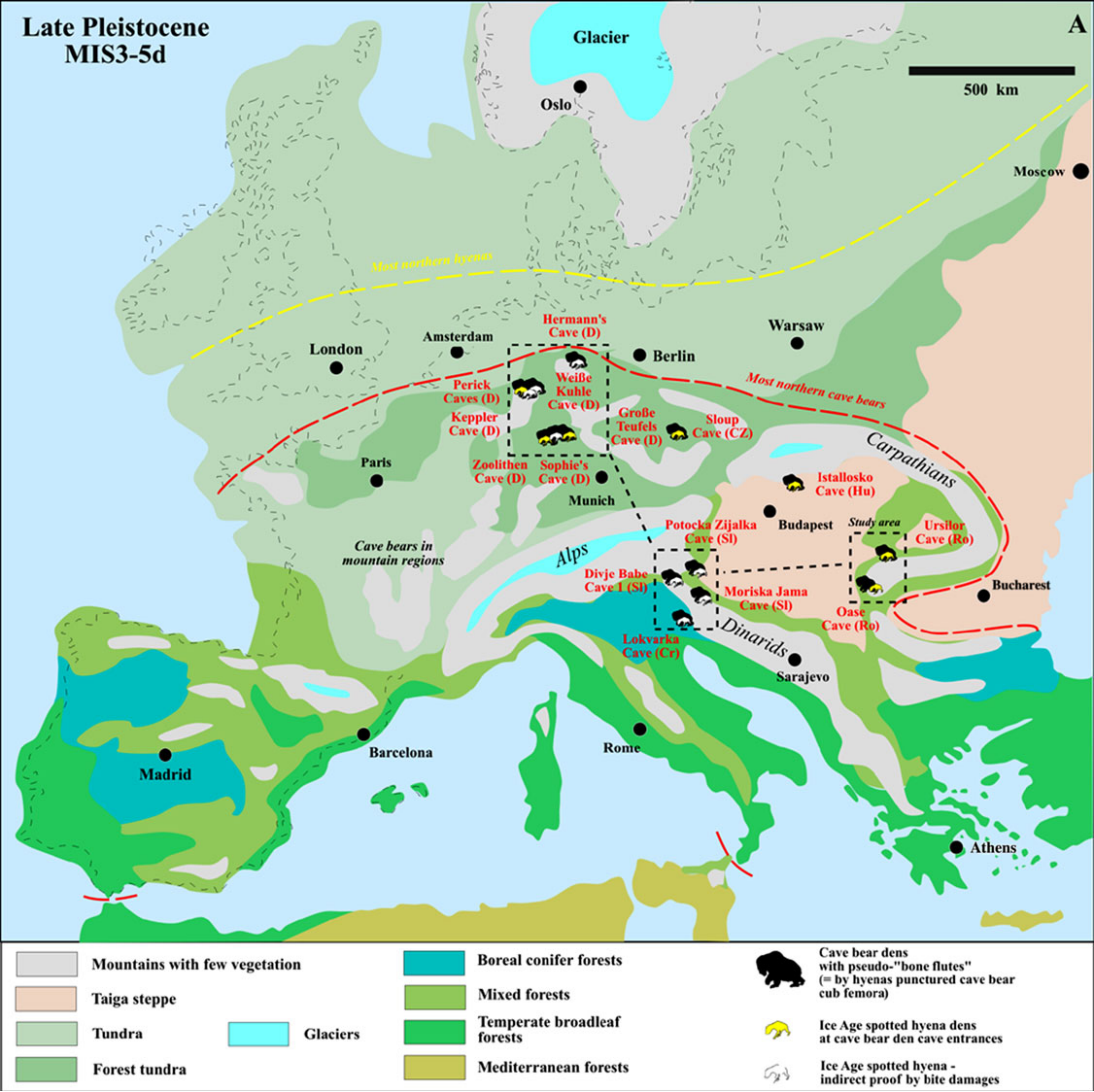
Studied and referred Late Pleistocene (MIS3–5d) European cave sites with ‘Palaeolithic cave bear pseudo-bone flutes’, and compared cave bear dens with hyena influence (hyena palaeobiogeography of 150 sites [4]).

The first ‘Neanderthal cave bear bone flute’ from the Middle Palaeolithic was believed to have been discovered in the 1920s from Potočka Zijalka Jama Cave (i.e. Potok Cave), Slovenia [1]. This was a larger cave bear den (cf. [2,3]) and Late Palaeolithic Aurignacian (not Neanderthal) used rock shelter camp site at the entrance (cf. [1]; figure 1). In this cave, cave bear hunts by Cro-Magnon humans seem to be indicated on a cave bear shoulder blade pathology (large diagonal impact hole, partly healed diagonal hole) that seems to have been made by a probable Mladeč projectile bone point [5].

Other cave bear cub femora with holes (‘bone flutes’) were then reported from the Istállóskö Cave, Hungaria (cf. [6]). This was a smaller cave bear and Ice Age spotted hyena (*Crocuta crocuta spelaea*) carnivore den which overlaps with another Aurignacian camp site, but again, it has no Middle Palaeolithic Neanderthal occupation signs (cf. [7]).

Brodar [8] reported cave bear cub femora and other cave bear bones ‘with holes’ as further proof of the ‘oldest instruments in the world’ from the Mokriška Jama Cave (or Medvedja Jama Cave=Bear Cave), Slovenia. Also, this is a large cave bear den which had again an Aurignacian camp site at the entrance, and again no Neanderthal occupation at all (cf. [9,10]).

**Figure 2.**
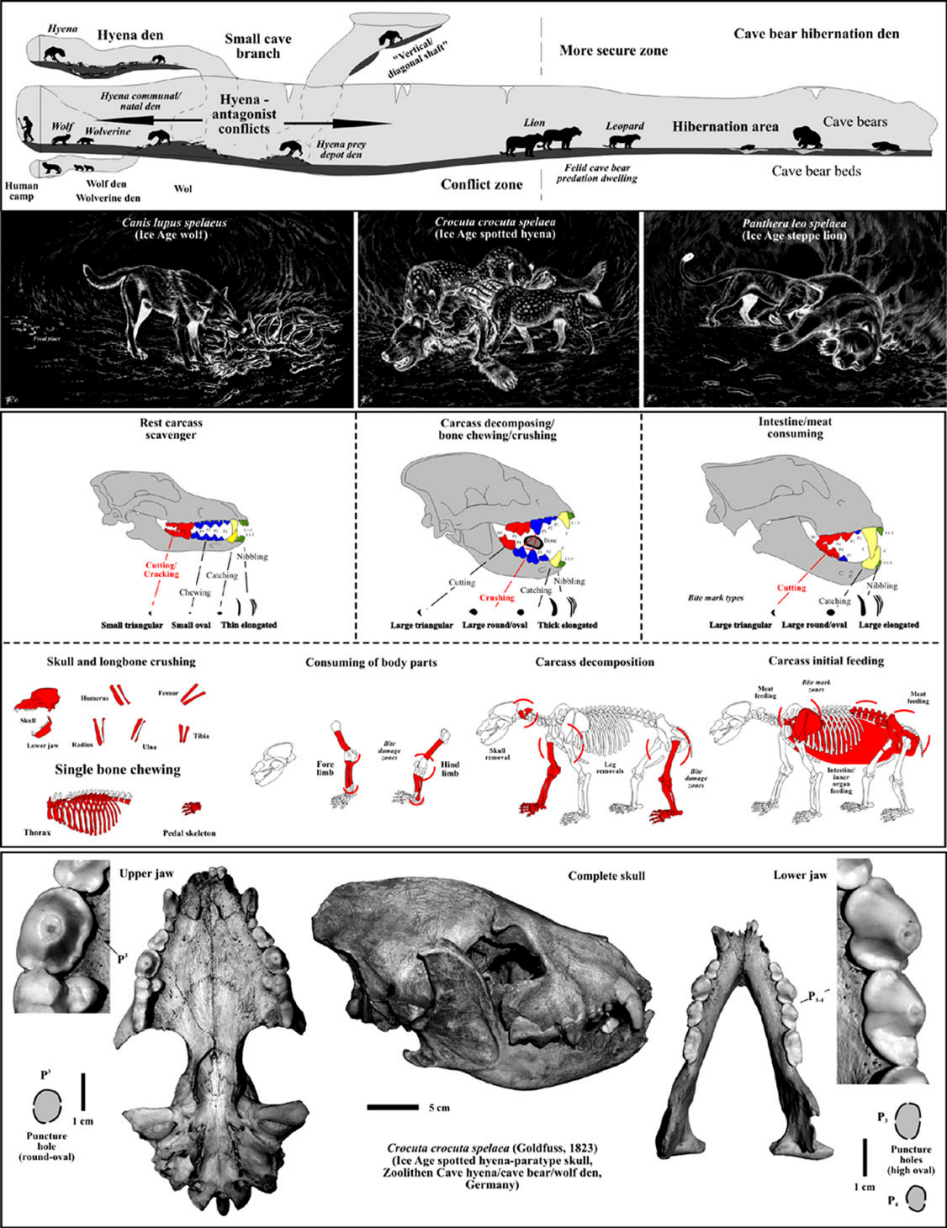
Cave bear scavenging models in larger cave bear den caves (here Zoolithen Cave, Germany) for all three top predators that hunted, killed and scavenged on cave bears all over Europe within caves in boreal forest palaeoenvironments. The bone crusher of longbones was only the Ice Age spotted hyena, which produced round/oval puncture marks on cave bear cub bones by the bone crushing premolar teeth, i.e. ‘bone flute holes’ (composed and adapted from [4,14,15,22,23]; illustrations G. Teichmann).

**Figure 3.**
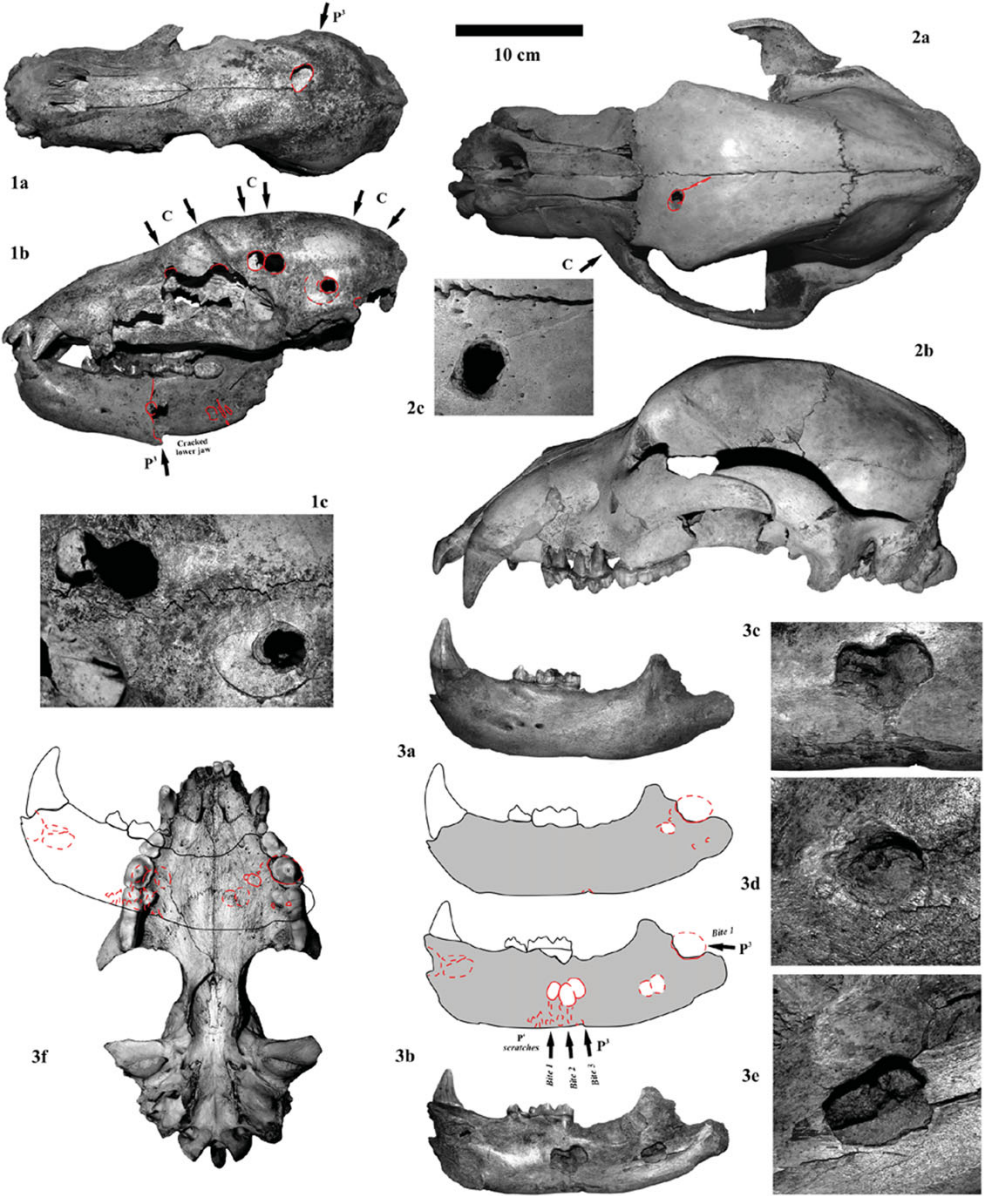
Carnivore puncture holes in cave bear skulls, jaws and postcranial bones caused by top predator canine teeth (lions, leopards, hyenas and wolves), but are mainly products at longbones and lower jaws of the premolar cracking teeth of hyenas (cf. figure 2). (1) Cub skull (small cave bear form *U. spelaeus eremus*) from the Weiße Kuhle Cave, Germany, which was scavenged strongly on the left side. Puncture holes are produced by canines (in cranium), whereas the breakage of the left mandible is the result of hyena premolar cracking teeth. (a) Dorsal, (b) lateral, (c) detail of lateral tooth mark holes (produced by carnivore canines, best fitting to hyenas or lions) (PAL collection). (2) Single probably canine impact of a large carnivore (lion, hyena) on a cub skull (large cave bear form *U. ingressus*) from the Große Teufels Cave, Germany. (a) Dorsal, (b) lateral, (c) detail of tooth mark hole (GTCP collection). (3) Mandible (*U. s. eremus*) from the Weiße Kuhle Cave of a cub with hyena premolar impact holes (cracking purpose). Such mandibles were crushed always similar with damaging the ramus, or flakes of the lower distal mandible. (a) Lateral outer view, (b) lateral inner view, (c–e) details of puncture holes of both sides and (f) refitting of the jaw with all tooth marks of both sides projected in one level which fit in one tooth mark of the bone crushing teeth of the upper jaw of a hyena (all PAL collection).

**Figure 4.**
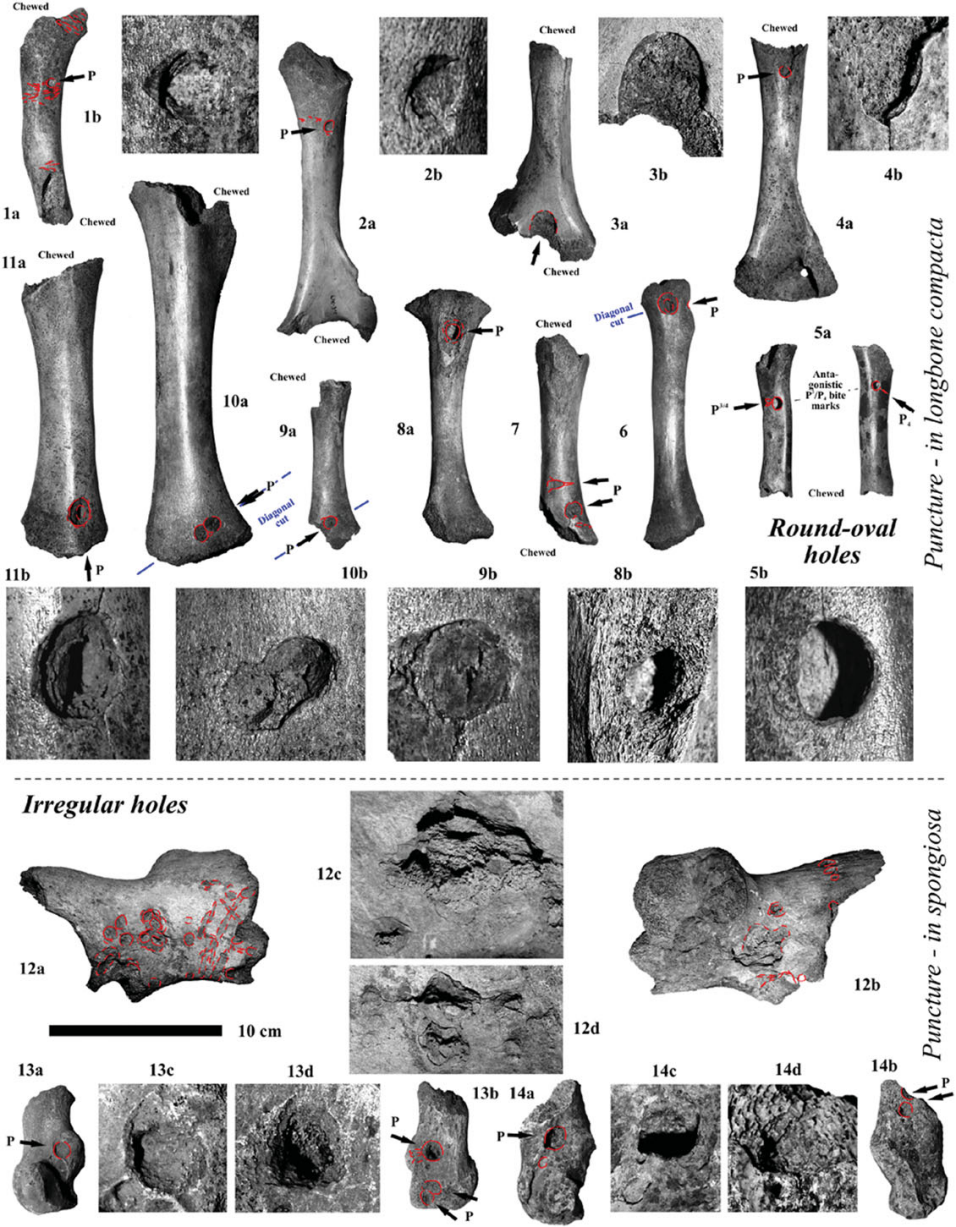
Carnivore puncture holes in cave bear (*U*. *s*. subsp. and *U. ingressus*) longbones (humerus, radius, tibia) and pelvic and pedal bones by top predator (lions, leopards, hyenas and wolves) canine and mainly premolar hyena teeth. (1–4) Cub humeri from the Weiße Kuhle Cave, Germany. (5–6) Cub radi from the Weiße Kuhle Cave, Germany. (7–11) Cub tibiae from the Weiße Kuhle Cave, Germany. (12) Cub coxa from the Weiße Kuhle Cave, Germany. (13–14) Cub and adult calcanei from the Weiße Kuhle Cave, Germany (all PAL collection).

**Figure 5.**
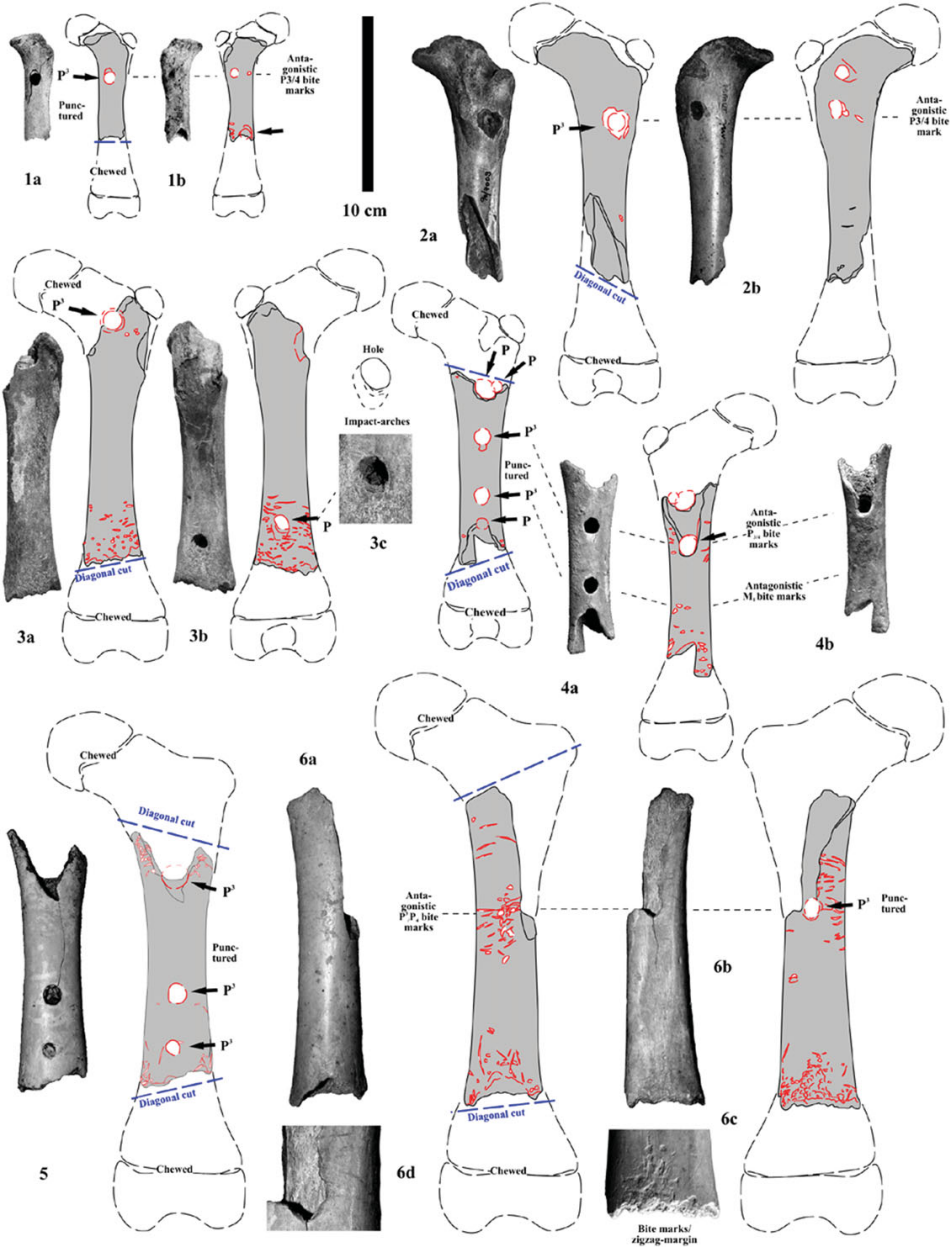
Pseudo ‘Neanderthal bone flutes’ of different aged cave bear (*U*. *s*. subsp. and *U. ingressus*) cub femora (less than 1 year individual age) from various European large cave bear den sites. (1) Femur from Mokriška Jama Cave, Slovenia (photos adapted from [24]; NMLS collection). (2) Femur from Keppler Cave, Germany (photos adapted from [4]; SMM collection). (3) Femur from Sophie's Cave, Germany (photos adapted from [22]; SMM collection). (4) Femur from Divje Babe Cave 1—‘the Neanderthal bone flute holotype’, Slovenia (photos from NMLS collection). (5) Femur from Oase Cave, Romania (IR collection). (6) Femur from Hermann's Cave, Germany (photos adapted from [16]; RC collection).

**Figure 6.**
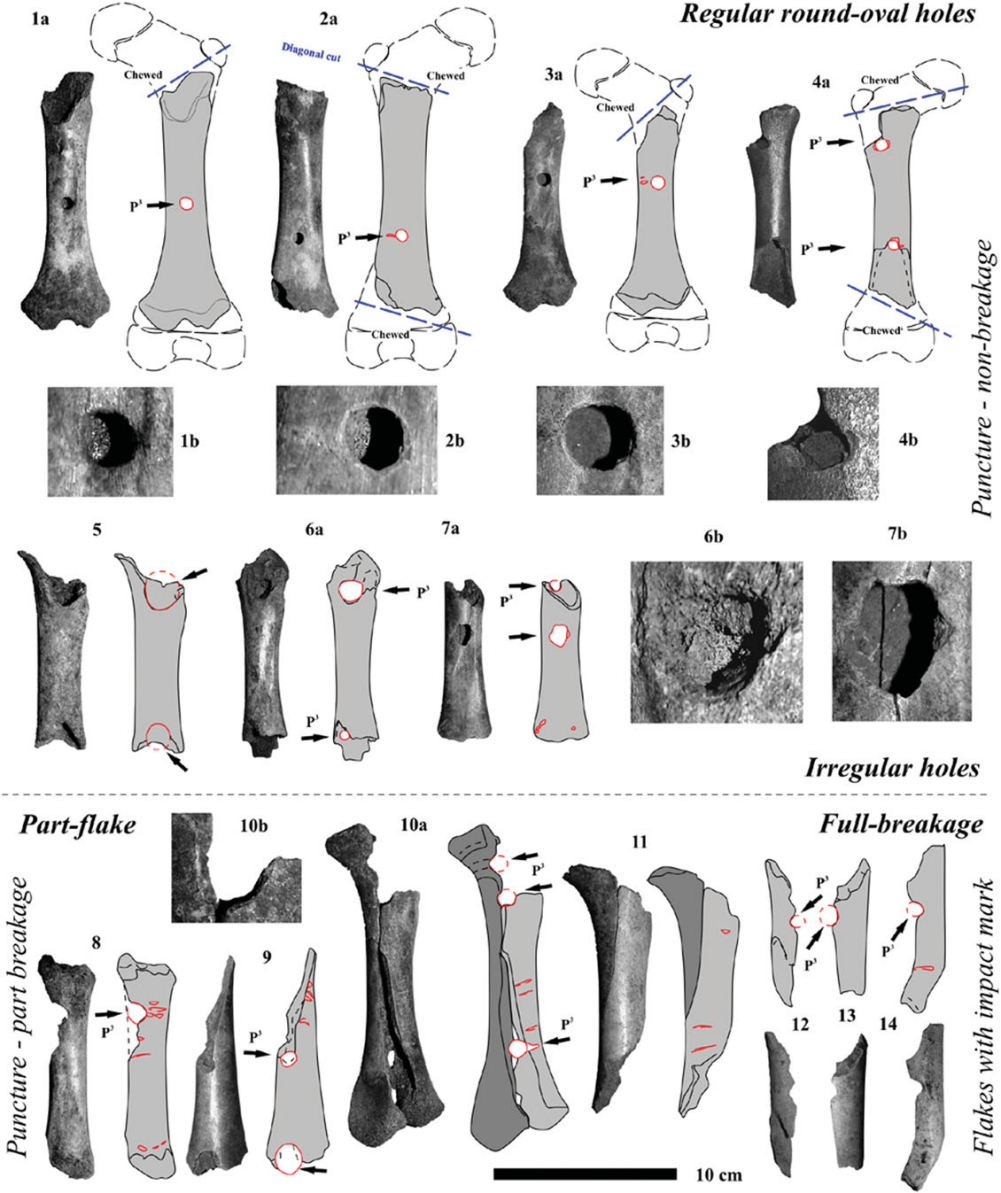
Continuous documentation of destruction stages of cave bear (*U*.*s*. subsp. and *U. ingressus*) cub femora: (1–7) puncture, (8–9) part-flake, (10–14) full breakage-flakes—all with puncture holes or half preserved holes after splitting in flakes—of different aged cave bear cub femora (less than 1 year individual age) and different species (*U. s. eremus* and *U. ingressus*)—all from the Weiße Kuhle Cave, Germany (PAL collection).

**Figure 7.**
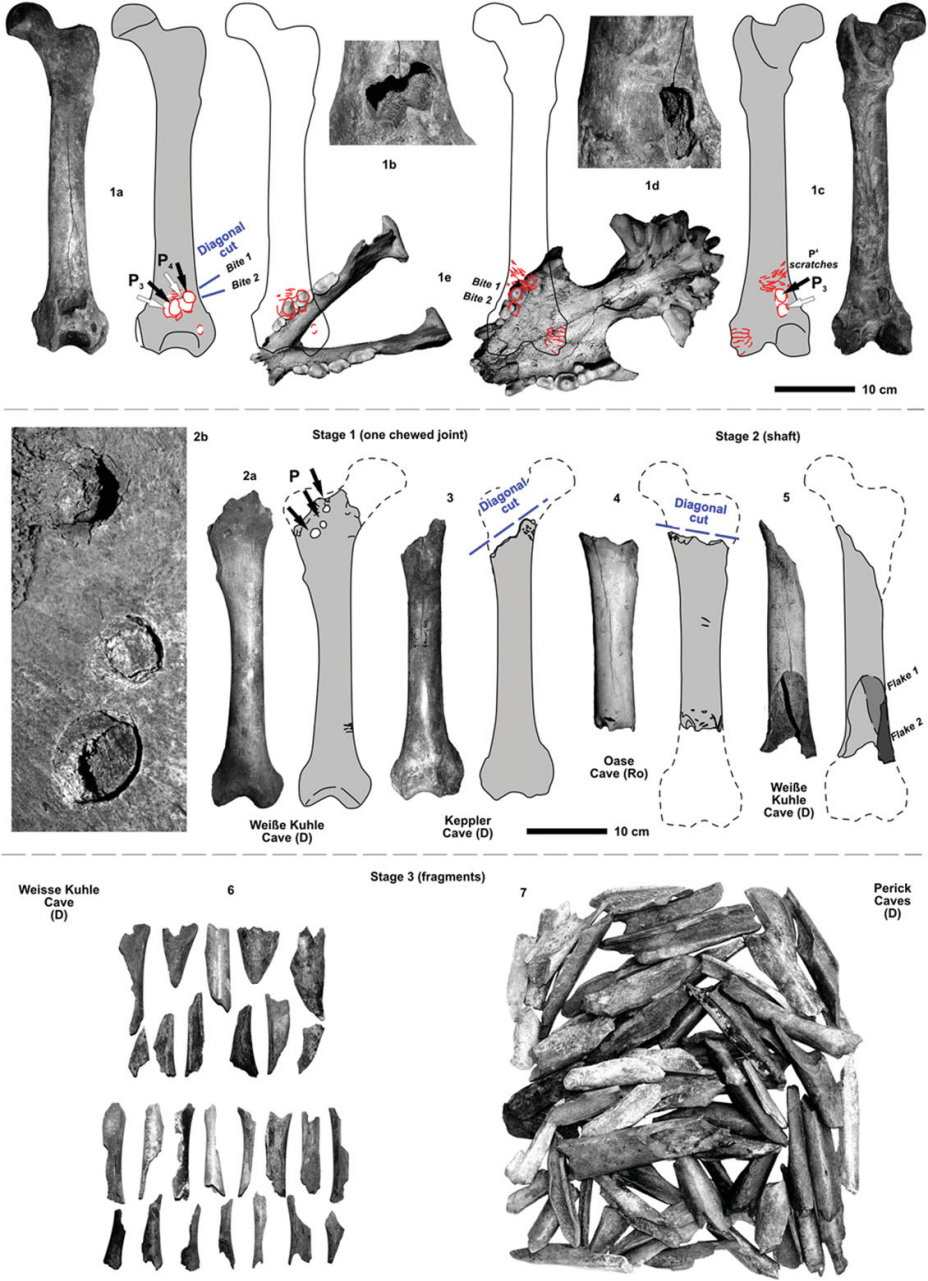
Examples of the destruction stages of femora of cave bear cubs, subadult to adult cave bears (*U*. *s*. subsp. and *U. ingressus*). (1) This femur of an adult cave bear (*U. s. eremus*) from the Große Teufels Cave, Germany (PO collection), is the best proof for the hyena tooth mark and damage origin, where two diagonal tooth marks (i.e. diagonal cut) can be reconstructed, and where lower and upper jaw premolar teeth and their antagonistic tooth mark impact holes fit exactly to the hyena skull dentition. A hyena tried to cut the distal joint. (a) Cranial view, (b) detail of the cranial tooth mark holes, (c) caudal view, (d) detail of the caudal tooth mark holes, (e) reconstruction refitting of the P-teeth into the cranial and caudal tooth pits, demonstrating exact fitting and two overlapping diagonal tooth marks (GTCP collection). (2) Proximally chewed and punctured femur joint of a subadult cave bear (*U. s. spelaeus* or *U. ingressus*) from the Weiße Kuhle Cave, Germany. The impact marks are two types: (a) full and deep into the spongiosa, i.e. tooth with intact crown tip); (b) round surface breakages of compacta, i.e. tooth with rubbed or damaged tip or slight impact (PAL collection). (3) Cut of proximal joint (*U. s. eremus*) demonstrated at a femur from the Keppler Cave, Germany, cranial (SMM collection). (4) Shaft from the Oase Cave, Romania, cranial (IR collection). (5) Shaft of a subadult (large cave bear *U. ingressus*) with distally cracked parts (all found in the cave close to each other with old fractures) from the Weiße Kuhle Cave, Germany, cranial (PAL collection). (6) Selected femur fragments of cub to subadult cave bears (*U. s. eremus* and *U*. *s*. subsp.) partly with spiral breakage, and tooth mark impact marks on the surfaces from the Weiße Kuhle Cave, Germany (PAL collection). (7) Many selected femur fragments of subadult to adult cave bears (*U. s. eremus* and *U*. *s*. subsp.) partly with spiral breakage, and tooth mark impact marks on the surfaces from the Perick Caves, Germany (PCH collection).

All aforementioned femora and other cave bear bones with ‘holes’ (i.e. ribs, humeri and jaws) were compiled and studied by the ethnologist/musician Omerzel-Telep [11], without any natural science, nor palaeozoology background, especially the important ecology of cave bears and their predators/scavengers, non-human top predators of the Ice Age and the wide distribution of cave bear den caves in Europe (cf. [3,12–21]; figures 1 and 2), where always large amounts of damaged and also punctured cave bear bones are present, such as figured with many new examples herein for the northern German Weiße Kuhle Cave and other cave bear dens (figures 3–7).

### 2.2 The long discussed Slovenian punctured cave bear cub bone find

Another juvenile bear cub femur with holes from Divje Babe I Cave, Slovenia, a small cave bear den (cf. [25]; figure 5(4)), where also Neanderthal Mousterian layers were believed to be present [26], was declared twice incorrectly as the ‘oldest instrument’, a 43 140 BP old ‘Neanderthal flute’ from layer 8 [26,27] (figure 5(4)). This was already contradictory to the results of the archaeological inventory that is well acceptably declared to be solely of, again, Cro-Magnon human Late Palaeolithic origin, and not of Mousterian (cf. [28]). The Aurignacian lithic material appears also together with cave bear remains [25]. Therefore, there is no evidence for a Neanderthal (Mousterian) context and the cave bear remains, which even occur in several older and younger Late Pleistocene layers (cf. [25]). The only absolute date was made solely on a cave bear bone, the ‘bone flute’, whose age would date into the Neanderthal or ‘cave bear den’ time period. This report of a ‘cave bear femur bone flute’ was not the ‘oldest’, neither historically, nor by stratigraphy. The bone's holes on the dorsal side appear not to line up, whereas on the ventral side another hole was declared as the ‘thumb hole’. The studies even thrilled up to ‘exact musical studies’ [29]. Fink [30,31] declared then to the top of this, without natural scientific studies, that the hole spacing matched a ‘diatonic scale sequence, among the most widespread scales known’—which underlines, also contradictory, that this is not of human origin. Ethnologist/musicians created then a wave of ‘cave bear bone instruments’ based solely on ‘holes in bones’ (compiled in [11]), from all kinds of carnivore punctured cave bear bones, even other than femora. Other authors doubted the ‘flute’ and human origin however (e.g. [32–38]) or were fighting for pro-arguments (e.g. [39,40]). At least, very correctly, the ‘holes’ were mostly discussed to be of ‘carnivore chewing damage’ origin (cf. [32,33,37,41]), whereas X-ray scans did not prove any ‘drill-scratches around the holes’ or any marks of stone tools on the bones, and left again the question of the hole origin open (cf. [42]). The exact carnivore was never estimated, even by newer and fully controversial studies by Turk *et al*. [24] that lack carnivore ecology knowledge, especially in tooth and jaw function of top predators. Ignoring the top predator bone damage on Ice Age animal bones, again the pseudo-bone flute was not only ‘confirmed’, even more bone flute finds were added by the same Slovenian author (cf. [43]), who misidentified: (a) the site occupation by Neanderthals, as those of Aurignacians [28], (b) the bone, by rotating it upside down (see [44]), the 180^°^ rotation of which is corrected herein (figure 5*a*), (c) the general bone taphonomy of cave bear bones, and (d) carnivore jaw functions, especially hyenas, correctly presented herein (figure 2).

### 2.3 Hyena and cave bear populations over Europe: specialized cave bear scavenges

In this contribution, not only sole carnivore damage can be demonstrated on all previously published ‘pseudo-bone flutes’, which were already revised in some cases [4,16] (figure 2). Herein, even more of such cave bear bones with holes can be added with focus only on the femora (figures 5–7), from German and Romanian cave bear den sites (therefore not limited to Slovenia at all, as mentioned by Turk *et al.* [24]; see figures 1, 5–7 and table 1). Their producer, a large carnivore, and the main scavenger/bone destructor of the Ice Age, the Ice Age spotted hyena *Crocuta crocuta spelaea*, will be discussed as the oval hole producer herein (figure 2), based on the intensive Late Pleistocene central European cave bear and top predator studies in and outside caves of the past years (e.g. [3,12–16–22,51,54,55]). This results in a different viewpoint of modern zooarchaeology, multiple animal/human use of larger cave systems and cave models (figure 2). The Ice Age top predator research in Europe focused these past years on hunting of cave bears in large cave bear dens. There, damage on cave bear bones is now well known and reported in several publications (e.g. [3,4,16,18–21,51,56]; figure 2). All former archaeological, ecological focus cave bear ‘bone flute’ studies forgot all four cave bear predators—steppe lions (*Panthera leo spelaea*), leopards (*Panthera pardus spelaea*), Ice Age spotted hyenas (*Crocuta crocuta spelaea*) and Ice Age wolves (*Canis lupus spelaeus*)—which are known now to be cave bear killers, and main consumers in mountain regions, where mammoth steppe megafauna were absent [4,18–21]. These predators specialized in consuming mainly (and especially in winter times during cave bear hibernation) cave bears in boreal forest mountain regions, but in different ways and with different impact on the carcasses and bone destruction (cf. figures 2 and 3). However, the main ‘bone destructor’ is known to be the European Ice Age spotted hyena [19] (figure 2), with cave bear bone damage first understood at the overlapping hyena den (cave entrance) and cave bear den of the Perick Caves [50–52], with newer proof at Sophie's Cave [21,22], and Hermann's Cave [16] or Zoolithen Cave [18] and herein best demonstrated and newly added for the Weiße Kuhle Cave (figures 3, 4, 6 and 7).

**Table 1.** Studied and from literature compiled cave bear, hyena, wolf den sites with pseudo-bone flutes (i.e. punctured cave bear cub femora), and overlap of Late Palaeolithic Aurignacian camp sites at the cave entrances, or cave bear hunt signs deep in caves. ‘Pseudo-bone flutes’ are not in Middle Palaeolithic archaeological, but of Late Palaeolithic and cave bear den context with large carnivore influence.

locality	age	animal/human use	large carnivores	references
Divje Babe Cave (Sl)	MIS 3–5d, including Aurignacian	small cave bear den/Aurignacian camp site	*P. l. spelaea, P. p. spelaeus, C. lupus* cf. *spelaeus*	[25,28,45–48]
Hermann's Cave (D)	MIS 3–5d, including Aurignacian	large cave bear den (*U. spelaeus* subsp.), short-term hyena den at entrance, Aurignacian cave bear hunting site deep in cave	*P. l. spelaea, P. p. spelaeus, C. c. spelaea, C. lupus* cf. *spelaeus*	[16,17]
Istallosko Cave (Hu)	?MIS 3–5d	cave bear den	?	[6,7]
Keppler Cave (D)	MIS 3–5d	large cave bear den (*U. spelaeus* subsp.), short-term hyena den, wolf den at entrance	*P. l. spelaea, C. c. spelaea, C. lupus* cf. *spelaeus*	[4,49]
Lukvarka Cave (Sl)	?MIS 3–5d	cave bear den	?	[11]
Moriska Java Cave (Sl)	?MIS 3–5d	cave bear den	?	[9,10]
Perick Caves (D)	MIS 3–5d	large hyena den at entrance (cub raising, communal den type), large cave bear den (*U. spelaeus* subsp./*U. ingressus*)	*P. l. spelaea, C. c. spelaea, C. lupus* cf. *spelaeus, Canis*	[4,19,50–52]
Potočka Cave (Sl)	MIS 3–5d, including Aurignacian	large cave bear den/Aurignacian camp site, cave bear hunting site	*P. I. spelaea, C.I.* cf. *spelaeus*	[1–3,5,53]
Sloup Cave (CZ)	MIS 3–5d	large cave bear den/hyena den at side branch	*P. l. spelaea, C. c. spelaea, C. lupus* cf. *spelaeus*	[54]
Sophie's Cave (D)	MIS 3–5d (?e)	large cave bear den/short-term hyena den at entrance	*P. l. spelaea, C. c. spelaea, C. lupus* cf. *spelaeus*	[18,19,21,22]
Große Teufels Cave (D)	MIS 3–5d	large cave bear den/short-term wolf and ?hyena den at entrance	*P. l. spelaea, C. c. spelaea, C. lupus* cf. *spelaeus*	[18,19], herein
Oase Cave (Ro)	MIS 3–5d	large cave bear den/Aurignacian skull find site	*C. c. spelaea, C. lupus* subsp.	[4]
Urşilor Cave (Ro)	MIS 3–5d (?e)	large cave bear den, short-term hyena den at entrance	*P. l. spelaea, C. c. spelaea, C. lupus* cf. *spelaeus*	[4,13]
Weiße Kuhle Cave (D)	MIS 3–5d	large cave bear den (*U. spelaeuseremus*/*U. ingressus*), ?short-term wolf den at entrance	*P. l. spelaea, C. lupus* cf. *spelaeus, P. p. spelaeus*, *Ursus arctos* subsp.	[4], herein
Zoolithen Cave (D)	MIS 3–5d (?e)	large cave bear den (*U. spelaeus* subsp./*U. ingressus*), hyena den, wolf den at entrance chamber area	*P. l. spelaea, P. p. spelaeus, C. c. spelaea, C. lupus* cf. *spelaeus*	[4,14,18]

## 3. Material and methods

First, from the literature available, ‘bone flutes’ were compiled and reinterpreted herein with new drawings (figure 5 and table 1). From the literature, new interpretations were made of the sites in the archaeological content (Neanderthal versus Aurignacian sites), and overlap in carnivore den use (hyena/wolf den—always at entrance areas) and identification as small to large cave bear dens (figure 1 and table 1). Several cave bear dens were studied, as well as larger bone collections (figure 1 and table 1). To those non-Slovenian/Hungarian (where bone flutes were thought only to be found) sites belong the Romanian Urşilor Cave and Oase Cave. German sites are Hermann's Cave, Perick Caves, especially a large population of small (*Ursus spelaeus eremus*) and large cave bears (*Ursus ingressus*) and large amount of material in different destruction stages from the Weiße Kuhle Cave, but also some relevant bones from the den sites Keppler Cave, Zoolithen Cave, Sophie's Cave, Große Teufels Cave and the Czech Sloup Cave.

General cave bear bone damage by large carnivores (lion, hyena, wolf) is present in all of those large cave bear dens. On average 80% of the cub, and 20% of the adult cave bear bones have large predator damage. The comparison focused on the presence/absence and positions of round–oval puncture marks. Herein the bone damage stages 1–3 (1, chewed joint; 2, shaft; 3, fragments) are presented in detail for cave bear femora of cubs, subadult and adult cave bears. This material is composed and compared from the aforementioned publications to end the long discussion about ‘Middle Palaeolithic Neanderthal bone flutes and oldest instruments’. Tooth mark types, shapes and especially their positions on both sides of the shaft ends, or the middle part, were identified as the antagonistic upper and lower jaw tooth marks of hyenas.

Furthermore, the available material was studied on the tooth mark margins and holes under a microscope, which allows identifying in a first step without reflection electron microscopy or X-ray photos possibly drill or stone tool scratch marks. No such cut/drill marks were found within the herein figured material. Instead, stone tool caused curved cut marks were found on a single cave bear femur (*U. ingressus*) from the latest Late Pleistocene (MIS3 cave bear layers, also Aurignacian period) of Hermann's Cave (cf. details in [23]). Other bone surface damages in the form of bites were observed on two cave bear cub humeri (*U. s. eremus*) from Sophie's Cave, but those were well identified to have resulted from porcupine (rodent) incisive teeth (cf. details in [21]).

Finally, the position of the bite mark holes and their orientation in oval pits are calculated at similar more or less cylindrical in the middle of the shaft formed longbones: radius, femur and tibia.

The figured ‘bone flutes’ of Divje Babe Cave 1, Mokriška Jama Cave and all other Slovenian ‘pseudo-bone flutes’ are housed in the National Museum in Ljubljana, Slovenia and Hungary (NMLS). Oase Cave material was studied in the Institute Emil Racovita, Romania (IR). Weiße Kuhle Cave (Germany) bones are in the PaleoLogic Research Institute, Czech Republic (PAL), Perick Cave material is kept in the collection of the Perick Cave club house in Hemer, Germany (PCH). Hermann's Cave material was analysed in the collection of the Rübeland show caves, Germany (RC), Keppler Cave material is in the Statdmuseum Menden, Germany (SMM), and Große Teufels Cave bones are kept in the show cave of the village of Pottenstein (PO).

## 4. Results

### 4.1 Bone damage stages: cub and adult differences

**Figure 8.**
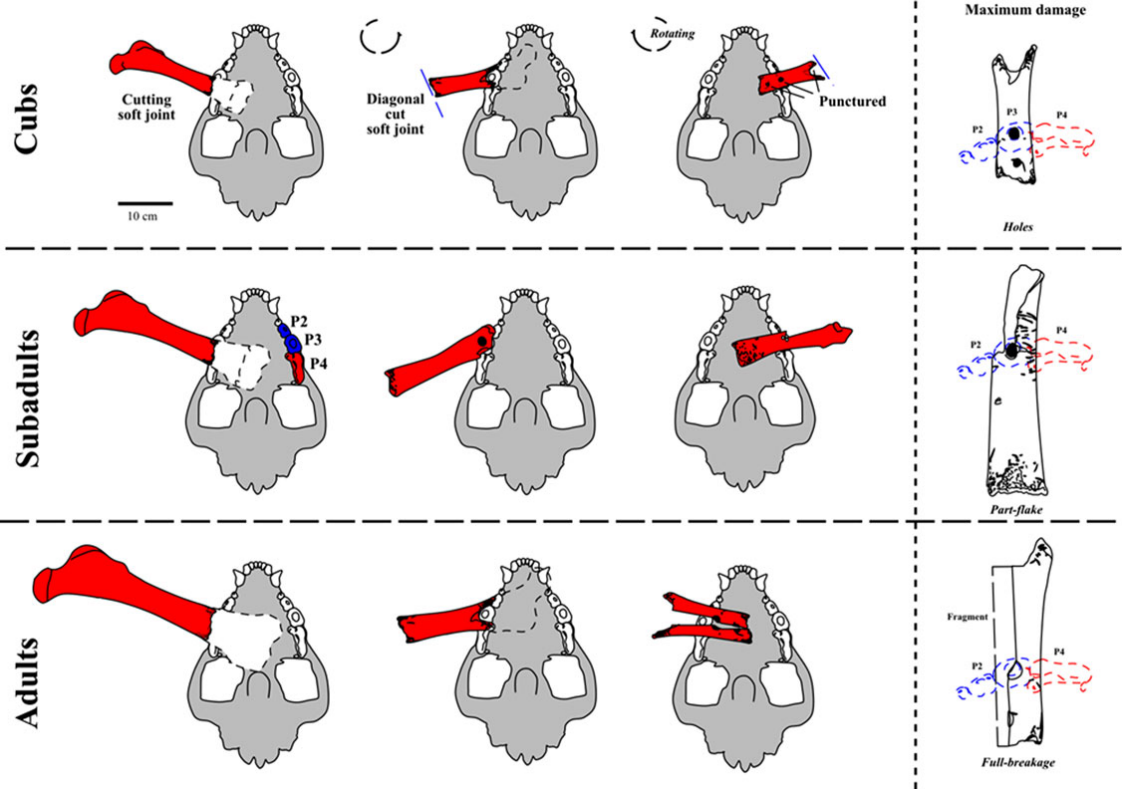
Stages of cave bear femur destruction by Ice Age spotted hyena. On cub femora, which are not well calcified and elastic-spongious in the compacta, hyenas produced in many cases only holes with their premolar bone crushing teeth (mainly P^3^) due to unsuccessful bone crushing (femur from Oase Cave, Romania). Subadult cave bear femora initially flaked (femur from Hermann's Cave, Germany). Adult femora have no puncture marks, because those directly flaked into pieces.

In a first stage, one of the joints (damage stage 1), and in a second step (damage stage 2) the other joint was cut off using the scissor-dentition on very small-sized femora (figure 5(1)), which becomes more diagonal (i.e. diagonal cut) with increasing femur sizes (figures 5–7). The larger the distal femur joint has been, the more diagonal this was cut. Cutting off the joints is recognized in all age classes of cave bears. Between damage stages 2 (cutting) and 3 (cracking), there are already differences in cub to adult cave bear femora (figures 6 and 7). Puncture marks of premolar teeth are only present in cub femur bones, owing to slight phosphatic calcification of the shaft. In some cases, these shafts expose, on both sides, puncture holes of each of the upper jaw P^3^ and antagonistic lower jaw P_3–4_, sometimes parts of M_1_, which attributes it only to the crushing teeth triangle of hyenas (cf. figures 2 and 3). In stage 3, subadult cave bear femora already started to crush, which is demonstrated from at least one example from Hermann's Cave (figure 5(6)). The detail continuous stages of cub femora puncture to breaking stages are demonstrated for the first time herein in the Weiße Kuhle Cave material (figure 6), whereas breakage is much rarer in subadult to adult femora (figures 6, 7 and 8). With increasing calcification of the shaft compacta, spiral breakage and sometimes back flaking patterns occur. Such bone fragments, here compiled for the Perick Caves (figure 4), do not expose any puncture marks of the premolars, generally, because the bone breaks are within the crushing triangle, and not by a puncture hole. Biomechanically the bone shaft (nearly conical cross section) cracks because of pressure on three sides (crushing triangle premolars). The main and strongest impulse of the crush comes from the most powerful upper P^3^. Sometimes, cave bear femora show smaller round–oval tooth marks, or on the shaft ends only half of the puncture mark is on the margin (e.g. Sophie's Cave and Divje Babe 1 Cave bear cub femora; figures 3(3) and 4). There are further arguments for the hyena origin on longbone shaft punctures found similar to radius and tibia cub shafts which are similar to femur shaft cross section, but stronger in their compacta in this animal age stage (figure 4).

### 4.2 Position of punctures and on bone types

The amount of bone material is still not enough to present clear statistics. However, with the herein used Weiße Kuhle material being very representative for a large cave bear den, puncture holes are found in the cave bear cub humerus (4×), ulna (0×), radius (2×), femur (13×) and tibia (5×). The position of the holes is mostly on the herein studied 19 cub femora, on the ventral side, and not on the dorsal convex side (cf. figures 5 and 6, and e.g. ‘bone flute holotype’ figure 5(4)). This area is thinner in the compacta than the dorsal one. In total, holes are ventral (7×), dorsal (3×) and in five cases on both sides. Those are the most important to understand their hyena tooth mark impact origin, because those can be attributed well to the upper and lower jaw antagonistic bone crushing premolar teeth (figures 5(1–6), 6(10) and 7(1)). Another argument comes from the oval holes, if attributed to the bone crushing premolar hyena teeth their elongation axes are in most cases parallel to the bone shaft, but only in holes within the shaft. At the shaft ends those vary more, and result from diagonal cuts, mainly. One more argument for the holes to be of hyena origin (or carnivore tooth mark in general) are the tooth mark hole margins. There are never signs of ‘drill marks on the margins of the compacta’, and in many cases there are breakages around the hole (i.e. impact circles, cf. figures 4–7). The final proof of holes in femur shafts comes from the crushed and flaked specimens (figure 6(8–14)), which are documented herein for the first time within a cave bear den. In two cases, old breakages are demonstrated on refitted femur shafts of cubs (figure 6(10–11)), and one subadult/adult shaft (figure 7(5)); in all cases the fragments have even different colours and were embedded after crushing in different sediment types/layers.

## 5. Discussion

### 5.1 Dating: Aurignacians versus Neanderthals

All ‘cave bear cub femora bone flute’ sites failed to date into the ‘Neanderthal times’ because all are not of Neanderthal (Middle Palaeolithic) human, but are instead from modern human Aurignacian occupations in ‘archaeological layers’ at entrances of cave bear dens, cf. [1,7,9], or deeper in caves due to cave bear hunt [23]. There, the cave bear layers themselves, which generally span from the MIS3–5d=25,000–113,000 BP, overlap/intercalate with the Cro-Magnon times, mainly Aurignacian, partly Gravettian, cultural layers [5,23,57]. Cave bear bones and archaeological layers are therefore not exactly isochronous in several cases (even mixed due to possibly bioturbation by cave bears building their nests, or burrowing porcupines or digging Ice Age spotted hyenas; cf. [13,20,21]), and ‘cave bear bone flutes’ would have been, if such, from modern human layers, in all cases.

### 5.2 Cave bear subspecies and ‘pseudo-bone flute origins’

The pseudo-bone flutes all come from layers of the MIS3–5d (herein added up to MIS 6) and are from smaller early cave bear forms of *Ursus spelaeus* subsp. (i.e. *U. s. eremus, U. s. spelaeus* *sensu* taxonomy of Stiller *et al*. [58]); interestingly though, alpine Late Pleistocene cave bear forms (*U. s. ladinicus*) do not show such holes in femora (i.e. indicator of absence of hyenas in alpine regions, and proof of holes made only by hyenas which are found only in middle high elevated mountain regions [19]). As is now well known, Aurignacian humans lived in Europe together with the last and largest cave bear species *U. ingressus* [16,18,21,23,58,59]. At a few cave sites in Europe, hunting of cave bears with propulsory spears is documented for the Aurignacians–Gravettians [5,22,23,57]. Therefore, the ‘pseudo-bone flutes’ originate from both smaller *Ursus spelaeus* subsp. (*eremus* or *spelaeus*) and the large *U. ingressus*, and from mountainous regions, where Ice Age spotted hyenas were around all over Europe (cf. map in [19]).

### 5.3 Stone tool drill experiments

Drilled holes were produced experimentally for a reconstruction of a ‘cave bear cub bone flute’ (cf. [60]) and are very different also on the hole margins and forms. All herein figured cub femora have, different from drill-holes, distinct characters (figures 5–7): (a) the holes are not fully round, instead oval-shaped, and beside the hole (see also [24]) a breakage-arch indicates an ‘impact’, rather than drilling (cf. also modern hyena impact mark pictures in [61]), (b) the margins are convex in cross-shape, and not steep-straight as with drills, (c) the corners are smooth and do not have drill/cut mark signs, at all, and (d) in most cases (figures 5–7), the antagonistic punctures/tooth marks (lower/upper jaw dentition fit) are present. Such antagonistic tooth marks are found often at different medium-sized hyena prey bones including their own species femora or even Neanderthal femora [19,20], also documented in the modern actualistic spotted hyena bone accumulation record [61–63]. Finally, also X-rays of the ‘bone flute’ hole margins did not verify any ‘drilling’ nor any stone tool work on the bone (cf. [64]).

### 5.4 Mice tooth marks and holes

Small parallel rectangular scratches on the pseudo-bone flute of Divje Babe Cave 1 were misinterpreted as cut marks (cf. [24]). Also one hole of the pseudo-bone flute of Istállóskö Cave is clearly produced, or a tooth mark hole extended, by mouse chewing (cf. photo in [6]).

### 5.5 Canine tooth mark impact hole ‘theories’

These are not present on the bone shafts, as fang teeth of hyenas (or any other carnivore) are never used to crack longbones (e.g. [61]). The dentition is very heterodont in those specialized mammals (cf. figure 2). The incorrect biomechanical illustration of the hyena teeth and jaw function leads to incorrect interpretations of hyenas as possible producers (cf. [24]), which attributed possible ‘holes’ to ‘canines’, which was contradictory to several arguments by Turk *et al.* [24], because the distal ends were already said to have ‘carnivore damage’ [44]. The tomography (cf. [65]) restudy of the bone excluded Ice Age hyenas, arguing with ‘abnormal biting or chewing behaviour using their canine teeth’ (cf. [66]). Indeed, neither hyenas, modern nor extinct, nor any carnivores mentioned use canines for ‘bone crushing’ (e.g. [22,61,67]; figure 2). Also, possibly most herbivorous small *U. s. eremus* and full herbivorous *U. ingressus* (e.g. [59]) cave bears did not use the canines for ‘bone crushing’, which were also proposed to be the ‘hole producers in cave bear bone shafts’ in former times, also forgetting the top predators of cave bears in the ‘cave bear book’ (cf. [68]). Some actualistic proof for the non-use of the canines can also be found in modern brown bear *U. a. arctos* prey taphonomy studies, where those did not crush any longbones on ungulate carcasses, whereas those might puncture softer spongiosa parts with their canines [69]. Microscopic analysis (cf. [27]) declared the femur of a young cave bear (‘bone flute’) probably to be pierced by the ‘bears themselves’ also uncritically repeated incorrectly by others (cf. [33,70,71]). These old ‘cave bear cannibalistic models’ were already revised, with many arguments not to be existent, including the top predators as bone damagers (including human bones) in Europe (e.g. [4,16,19–21,72]). Including also the new studies of the omnivorous brown bears of Arilla *et al.* [69], those seem to exclude even cannibalism within *U. a. arctos.* Also the figure of bone crushing by Turk *et al.* [24] was incorrectly presented, using only one lower jaw premolar, although the bone crushing triangle consists of three teeth (figure 2). Using all these strange presentations by Turk *et al.* [24], this indeed would be abnormal for hyenas to try to crush longbones with their canine teeth. Which hyena (lion and wolf) teeth are responsible for what kind of tooth marks and bone damage on cave bear bone femora and other few selected cranial and postcranial material is refigured composed of several studies [18–22] (figure 2).

### 5.6 Hyenas at cave bear dens in Europe

It is no wonder then that further incorrectness about cave bear bone taphonomy at Divje Babe Cave 1 was published (cf. [73]), because all ‘fragmented’ bones were simply declared as due to ‘sediment pressure’. Indeed, some are naturally weather-cracked. A studied ulna of a cave bear at the site is one of the best examples of bone crushing by hyena premolar teeth. Even the puncture marks in the upper shaft area are visible, demonstrating the scavenging/bone cracking activities also in the Divje Babe Cave 1, similar to that found in German caves (cf. [4,16,18,23]; cf. figures 5–7). His final arguments that ‘hyenas are absent’ at this site (cf. also carnivore fauna in [25]) are none, because as ignored in intensive cave bear den cave site taphonomy studies of Europe, the models of presence and absence of any large predator are well known [4,13,18,19,23]. Hyenas and other carnivores are rarely found at the ‘scavenging sites’, including caves and cave bear dens, because they are only found there when they occupied the cave entrances as (a) cub raising, (b) communal or (c) prey depot dens (cf. definitions and discussions in [4,14,18–20,20,21,54,74].

## 6. Conclusion

The ‘cave bear cub femora with holes’ are, in all cases, neither instruments nor human made at all. All cave bear pseudo-bone flutes are not dated to Neanderthal Middle Palaeolithic Mousterian layers, but instead, if possible to date, to Late Palaeolithic, Aurignacian/Gravettian layers. There, where they are dated absolutely (Divje Babe Cave 1) are without archaeological context at all, and simply of cave bear den use during the MIS 3–5d. At these times, different cave bear subspecies *Ursus spelaeus* subsp. *eremus* (smallest cave bear) and *spelaeus* (i.e. Neanderthal times) and *U. ingressus* (largest cave bear, i.e. Aurignacian/Gravettian times) used caves all over Europe for cub raising and hibernation. All the large carnivore punctured cave bear cub femora (and other punctured bones) appear always in small to large cave bear den cave/cave entrance contexts. This sometimes overlaps with hyena dens and human camp sites at cave entrances only, where cave bear den, carnivore den and human remains are even mixed up (partly separated in layers), all over Europe due to competition for and seasonal use of cave entrances/rock shelters. The cave bear bones with round–oval, larger puncture marks can be well attributed solely to the main cave bear scavenger of Europe—the Ice Age spotted hyena *Crocuta crocuta spelaea*. This main Late Pleistocene bone destructor in Europe is known recently with more than 150 den sites (95% are cave sites) all over Europe. At cave bear dens hyenas left, by periodic scavenging, up to 20% of damaged bones, whereas also lions (cave bear killers), leopards and wolves played a larger role in the cave bear hunting/scavenging, even deep in caves. Those indeed also left, in some cases, round–oval, larger punctures in cave bear bones, but with their canines only in soft spongiosa (pelvis, vertebrae), and never in any bone shaft compacta. Neither carnivores nor cave bears (herbivorous) used their canine teeth to crush longbones, or any other bones. Therefore, all other top predators—except hyenas—can be excluded, at least for the round–oval punctures in cave bear longbone shafts. Only hyenas have developed a carcass destruction and butchery strategy, also for cave bears. This strategy is demonstrated, herein in detail, on cave bear femora destruction (especially material from Weiße Kuhle Cave, Germany), which is presented in three stages and for different aged individuals—cubs (less than 1 year), subadults (1–2 years) and adults. Cub bones are ‘soft’ and thin-walled in the bone shaft compacta, which increases in subadults to adults. This explains why puncture marks are found only in cub (less 1 year) femora, and partly in subadults, whereas they are absent completely in adults, because hyenas cracked those bones into pieces with the premolar triangle teeth (i.e. bone crushing teeth) for access to the bone marrow and easier swallowing of those pieces for the bone collagen use. Hyenas left, therefore, ‘pseudo-bone flutes’ during the Late Middle to Late Pleistocene all over Europe in cave bear dens, and on different cave bear species/subspecies. This is known due to lack of breakage on most of the cave bear cub femora, which generally show additional diagonal zigzag margins (from chewing joints by scissor teeth of hyenas) or have triangular or smaller scratch tooth marks. This even allows reconstruction, in some cases in detail, the tooth mark of the upper and lower jaw teeth of hyenas—the last tooth mark of the premolar bone crushing triangle of the powerful jaws of the last hyenas of Europe. Finally, some flakes and refitted cub femora, both with tooth mark holes, prove the bone cracking activities at cave sites.
